# European experience of patients with HER2-positive advanced/metastatic breast cancer accessing trastuzumab deruxtecan through a named patient program: the EUROPA T-DXd study

**DOI:** 10.3389/fonc.2025.1650981

**Published:** 2025-10-14

**Authors:** Cristina Saura, Hans Wildiers, Giampaolo Bianchini, José Ángel García-Saenz, Arthur Allignol, Amanda Logue, Markus Lucerna, Sabine Stamenitis, Michelino De Laurentiis

**Affiliations:** ^1^ Medical Oncology Department, Vall d’Hebron University Hospital and Vall d’Hebron Institute of Oncology (VHIO), Barcelona, Spain; ^2^ Department of General Medical Oncology, University Hospitals Leuven, Leuven, Belgium; ^3^ Department of Medical Oncology, IRCCS Ospedale San Raffaele, Milan, Italy; ^4^ Department of Medical Oncology, Vita-Salute San Raffaele University, Milan, Italy; ^5^ Medical Oncology Department, Hospital Clínico San Carlos, Instituto de Investigación Sanitaria San Carlos (IdISSC), CIBERONC, Madrid, Spain; ^6^ Data and Statistical Sciences Centre for Real-World Evidence (RWE) and Evidence Generation (EG), Daiichi Sankyo Europe GmbH, Munich, Germany; ^7^ Global Medical Affairs, Evidence Generation & External Alliances, Oncology Outcomes Research, Oncology Business Unit (OBU) Medical, AstraZeneca, Cambridge, United Kingdom; ^8^ Medical Affairs Oncology, Daiichi Sankyo Europe GmbH, Munich, Germany; ^9^ Clinical Trials Management & Resources, Global Oncology Medical Affairs, Daiichi Sankyo Europe GmbH, Munich, Germany; ^10^ Department of Breast Oncology, Istituto Nazionale Tumori IRCCS Fondazione G. Pascale, Naples, Italy

**Keywords:** real-world data, antibody-drug conjugate, trastuzumab deruxtecan, metastatic breast cancer, HER2-positive

## Abstract

**Introduction:**

In January 2021, trastuzumab deruxtecan (T-DXd) received conditional approval in the European Union for the treatment of human epidermal growth factor receptor 2–positive (HER2-positive) unresectable or metastatic breast cancer in patients who had previously received two or more prior anti-HER2–based regimens. In March 2021, a named patient program (NPP) was initiated to enable eligible patients to access T-DXd when not yet locally available. This European, multicenter, multinational, observational, single-arm data collection study included heavily pretreated patients with HER2-positive metastatic breast cancer who received T-DXd under the NPP and was intended to generate real-world insights from routine clinical practice.

**Methods:**

Patients with unresectable or metastatic HER2-positive breast cancer who had received ≥2 prior anti-HER2–based regimens and were treated with T-DXd (5.4 mg/kg) under the NPP (DS8201-0002-EAP-MA) were eligible for inclusion in the study. Participation in the data collection was optional and independent of eligibility for the NPP. The primary endpoint was real-world time to treatment discontinuation. Secondary endpoints included real-world progression-free survival, prior HER2-targeted treatment patterns, reasons for T-DXd treatment discontinuation, safety, and antiemetic prophylaxis prior to T-DXd initiation. Adverse events were collected via a pharmacovigilance system.

**Results:**

In total, 256 patients (from centers across Ireland, Italy, and Spain) participated in the study. At data cutoff (March 28, 2024), 243 patients (94.9%) had discontinued treatment. The primary endpoint of median (95% confidence interval [CI]) real-world time to treatment discontinuation was 13.0 (11.2, 15.2) months. Median (95% CI) real-world progression-free survival was 15.2 (11.9, 17.3) months. The median number (range) of prior anti-HER2 lines of therapy in the metastatic setting was 3 (0–6). The main reason for T-DXd treatment discontinuation was disease progression (46.1%). Use of an antiemetic regimen with prophylactic intent was reported in 80.9% of patients. No new safety signals were identified.

**Conclusion:**

Results from this real-world study are consistent with the clinical benefit observed with T-DXd in patients with HER2-positive metastatic breast cancer in phase II/III clinical trials in the third-line setting and beyond.

**Clinical trial registration:**

https://clinicaltrials.gov/study/NCT05458401; identifier NCT05458401

## 1 Introduction

Breast cancer remains the leading cause of cancer mortality in women (1), accounting for 15.4% of female cancer-related deaths (1, 2). Human epidermal growth factor receptor 2 (HER2)–positive breast cancer, defined as HER2 gene amplification and/or protein overexpression, occurs in approximately 20% of primary breast cancers and corresponds to a clinically aggressive subtype (3, 4). Historically, HER2-positive breast cancer was associated with higher rates of disease recurrence and mortality (4). HER2-directed therapy is considered to be the gold standard of treatment for patients with HER2-positive breast cancer, and has been shown to improve responses and clinical outcomes compared with previous standard-of-care therapies (5–7). The administration of two anti-HER2 agents with different mechanisms of action, trastuzumab and pertuzumab, in combination with a taxane (THP), is the recommended first-line therapy for HER2-positive metastatic breast cancer, based on the results of the CLEOPATRA clinical trial (8–10). Prior to recent advances in the treatment of HER2-positive metastatic breast cancer, a HER2-directed antibody-drug conjugate (ADC), trastuzumab emtansine (T-DM1), was recommended as second-line therapy following progression during or after THP (5, 10–13).

ADCs are composed of an antibody coupled to a chemotherapeutic agent (payload) via a linker, and several newer ADCs have been developed since the approval of T-DM1, including trastuzumab deruxtecan (T-DXd) (14, 15). T-DXd is an ADC that contains a humanized immunoglobulin G1 monoclonal antibody specifically targeting HER2 (trastuzumab), a tetrapeptide-based cleavable linker, and a potent topoisomerase I inhibitor payload, deruxtecan (DXd) (14, 15). In January 2021, T-DXd was conditionally approved in the European Union for use as monotherapy in adult patients with HER2-positive unresectable or metastatic breast cancer who have received two or more prior anti-HER2–based regimens, based on results from the single-arm phase II DESTINY-Breast01 trial, which evaluated patients who had received previous treatment with T-DM1 (16–18). These data were subsequently confirmed in the randomized, open-label, multicenter phase III DESTINY-Breast02 trial, which evaluated T-DXd versus treatment of physician’s choice in patients previously treated with T-DM1 (19).

In July 2022, the European Commission extended the conditional marketing authorization of T-DXd to adult patients with HER2-positive unresectable or metastatic breast cancer who have received one or more prior anti-HER2–based regimens, based on the results from the phase III DESTINY-Breast03 trial (20–24). The trial demonstrated the superior efficacy of T-DXd over T-DM1 (median progression-free survival 28.8 months versus 6.8 months, respectively) (24). Patients treated with T-DXd also demonstrated improved overall survival compared with patients treated with T-DM1 (24, 25). Based on these results, clinical practice guidelines were updated to recommend T-DXd as the new standard second-line therapy in patients with HER2-positive metastatic breast cancer who have been previously treated with trastuzumab and a taxane (10).

At the time of writing, T-DXd is approved in more than 80 countries worldwide (26). However, despite the impressive results observed in clinical trials, more real-world evidence (RWE) about the effectiveness and safety of T-DXd in routine clinical practice is needed. In addition to their primary goal of providing access to treatment, named patient programs (NPPs) enable real-world data (RWD) collection and the generation of associated RWE. RWE is becoming increasingly valuable for regulatory and reimbursement purposes (27, 28). In March 2021, following conditional approval of T-DXd in the European Union, an NPP was initiated to enable eligible patients with an unmet medical need to access T-DXd when it was not yet available locally, either commercially or through an appropriate clinical trial. To gain insights on the use of T-DXd outside of a trial setting, European centers that had patients with HER2-positive metastatic breast cancer who were receiving treatment (or were previously treated) with T-DXd under the NPP were invited to participate in an RWD collection. The *EU*ropean *R*eal-world experience *O*f *P*reviously treated advanced/metastatic HER2-positive breast cancer patients *A*ccessing trastuzumab deruxtecan (EUROPA T-DXd) study investigated the effectiveness and safety of T-DXd in a heavily pretreated (third line and beyond) real-world population with HER2-positive metastatic breast cancer at sites in Spain, Italy, and Ireland. The primary objective of EUROPA T-DXd was to evaluate the real-world time to treatment discontinuation (rwTTD) under the NPP.

## 2 Materials and methods

### 2.1 RWD collection design

EUROPA T-DXd (NCT05458401) was a multicenter, multinational, observational, single-arm, data-collection study investigating the use of T-DXd in routine clinical practice for the treatment of adult patients with advanced or metastatic HER2-positive breast cancer who had received two or more prior anti-HER2–based regimens, and represents the collection of a limited set of data in the NPP ‘Trastuzumab deruxtecan’. Each participating physician’s decision to treat an individual patient was made prior to, and independent of, participation in RWD collection. T-DXd was administered under the NPP; the recommended dosage was 5.4 mg/kg intravenously every 3 weeks until disease progression or unacceptable toxicity. To manage adverse reactions, temporary interruption, dose reduction, or discontinuation were allowed. Patients were required to discontinue treatment under the NPP and switch to a commercial supply when T-DXd became available in their country.

Physicians submitted baseline and ‘on treatment’ RWD through a customized Zelta™ electronic case report form (eCRF); baseline was defined as the time period from the date of initial breast cancer diagnosis to the date of T-DXd treatment initiation under the NPP, and ‘on treatment’ was the time between the date of T-DXd initiation and either T-DXd discontinuation under the NPP or the end of the RWD documentation period (a target of 14 months after T-DXd initiation under the NPP), whichever occurred first. When treatment discontinuation was for a reason other than death, progression, withdrawal of consent for data collection, or commercial availability of T-DXd, follow up continued until disease progression or the closure of the RWD documentation period, whichever occurred first. Data were sourced from patients’ medical records and routine medical visits and recorded in the eCRF by the treating physician or designee (according to standard clinical practice and documentation in the treatment of the patient). To account for patients who had discontinued treatment prior to consenting to participate in the RWD collection, an option to collect all data retrospectively was built into the study design. For patients whose treatment remained ongoing at the time of providing consent, baseline data were entered retrospectively; discontinuation and follow-up data were collected prospectively, at the relevant timepoint.

### 2.2 Patient population

Adult patients were eligible if they were treated through ‘Trastuzumab deruxtecan’ (the NPP) for unresectable or metastatic HER2-positive breast cancer and had received two or more prior anti-HER2–based regimens. HER2-positive tumor status was determined by a validated method and defined as an immunohistochemistry (IHC) score of 3+ or a score of 2+ with positive fluorescence by *in situ* hybridization (ISH). Alternative treatment routes (including recruitment into a T-DXd clinical trial) were required to have been exhausted prior to enrollment. All patients (or a patient’s legally acceptable representative) provided written informed consent, or a waiver of informed consent granted by the Institutional Review Board/independent ethics committee, before participation in the study.

### 2.3 Objectives and endpoints of RWD collection

The primary objective of data collection was to evaluate the rwTTD, defined as the time from T-DXd initiation in the NPP to discontinuation for any reason in patients with HER2-positive metastatic breast cancer in a real-world clinical setting. The secondary objectives were to describe the following in a real-world clinical setting: prior HER2-targeted treatment patterns; reasons for T-DXd treatment discontinuation; adverse events (AEs); prophylactic treatment for nausea and/or vomiting prior to T-DXd initiation; and real-world progression-free survival (rwPFS), defined as the time from T-DXd initiation to the earliest of death or progression. Overall survival was included as an exploratory endpoint.

### 2.4 Safety reporting

To comply with pharmacovigilance legislation, physicians treating patients under the NPP reported serious and non-serious AEs and/or safety information using an AE report form and targeted questionnaires through a pharmacovigilance system. The reporting of AEs was not entered into the database via the eCRF; however, the participating centers were required to follow local laws and regulations for the spontaneous reporting of AEs during daily routine practice for patients treated under the NPP. As such, the collection of treatment-emergent adverse event (TEAE) data via the NPP included standard terms from the Medical Dictionary for Regulatory Activities (MedDRA). Any terms captured in the NPP that related to disease progression and metastases were omitted from this report.

### 2.5 Statistical methods

For the primary objective, rwTTD, sample size calculations were based on simulations that assumed a) an exponential distribution, b) a median time to treatment discontinuation of 10, 14, and 18 months, and c) a uniform T-DXd initiation period, in which patients may have initiated treatment between the start of the NPP (March 1, 2021) and up to 14 months before the closure of the RWD collection. Each scenario was repeated 10000 times. The simulation showed that a sample size of 200–300 patients would provide an acceptable level of precision. All centers with patients actively treated or previously treated with T-DXd under the NPP were invited to take part in RWD collection (contingent on local regulations permitting RWD collection). In each country, centers were ranked based on the number of eligible NPP patients at the start of data collection and were invited to take part in descending order (highest to lowest) until the planned sample size was reached.

All analyses were conducted on the full analysis set, which was analogous to the safety analysis set, and included all patients enrolled in RWD collection who received at least one dose of T-DXd. For the primary endpoint, rwTTD, the Kaplan-Meier method was used to estimate survival distribution, and the results were presented graphically. Estimates of medians and their corresponding 95% confidence intervals (CIs) were derived using the Brookmeyer and Crowley method. Patients still under treatment at the time of data cutoff and patients switching to commercial supply were censored at the relevant timepoint.

Descriptive statistics were used for analysis of the following secondary endpoints: prior treatment patterns, the reason for T-DXd discontinuation, and prophylaxis provided to patients for nausea and/or vomiting prior to T-DXd initiation. For the secondary endpoint of rwPFS, the distribution was estimated using the Kaplan-Meier method and presented graphically, with medians and their corresponding 95% CIs estimated using the Brookmeyer and Crowley method. Data were also analyzed in selected subgroups based on disease characteristics at baseline. A sensitivity analysis was performed whereby discontinuation for reasons other than progression or death were considered as competing events. The probability of progression or death was estimated using the Aalen-Johansen estimator of the cumulative incidence function (29).

For all time-to-event analyses, patients who switched to a commercial supply of T-DXd were censored at the last date of their participation in the NPP. Patients who withdrew consent for data collection were censored at the earliest of date of discontinuation or date of last routine medical visit.

## 3 Results

### 3.1 Patient demographics and baseline disease characteristics

**Figure 1:**
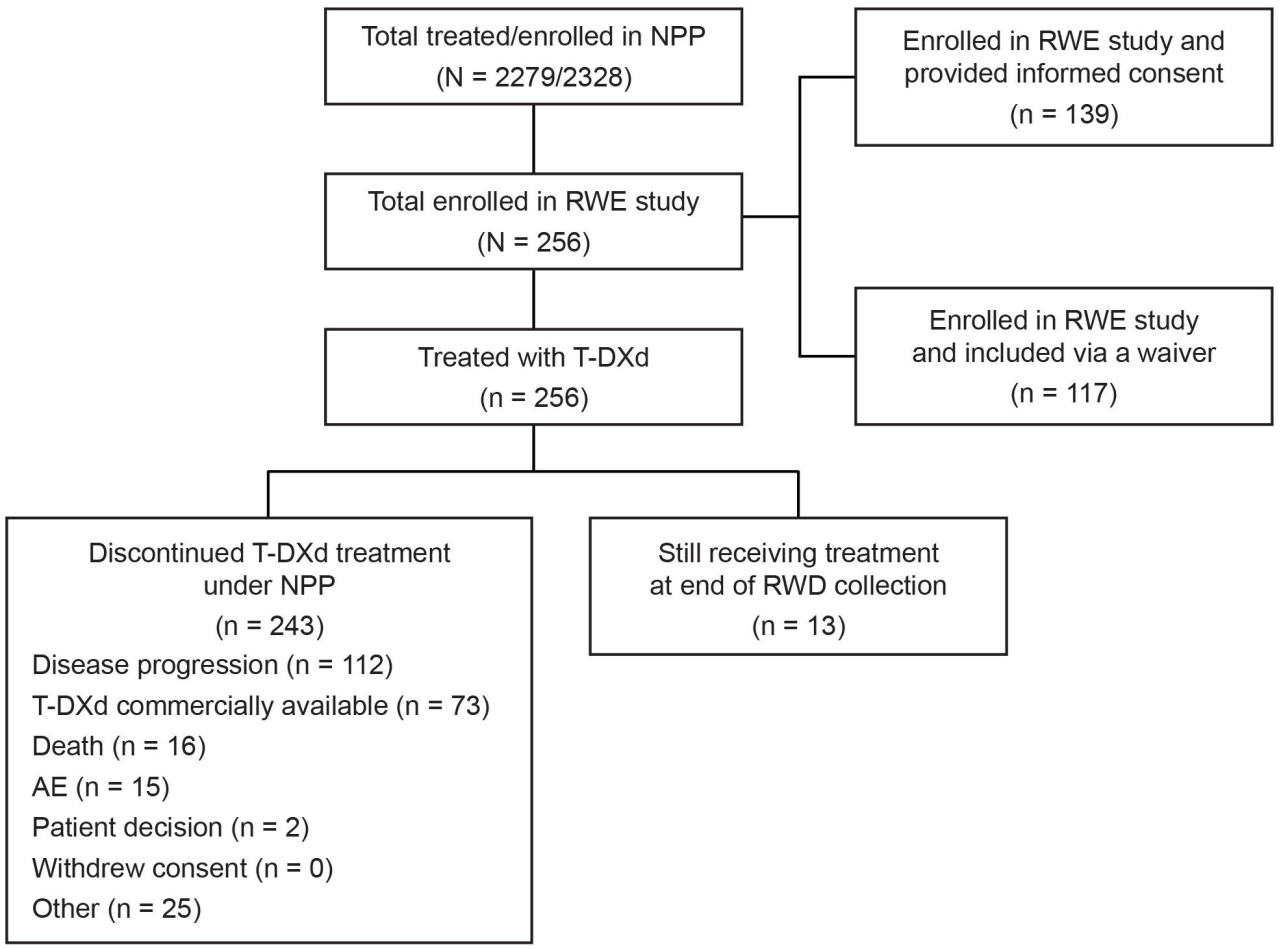
Patient flow and disposition. AE, adverse event; NPP, named patient program; RWD, real-world data; RWE, real-world evidence; T-DXd, trastuzumab deruxtecan.

Between November 22, 2022, and December 1, 2023, 20 centers in three countries enrolled 256 patients in total in the EUROPA T-DXd study (Italy, n = 172; Spain, n = 54; and Ireland, n = 30), as summarized in **Figure 1**. All enrolled patients received T-DXd under the NPP.

The demographic and clinical characteristics of the patients who received T-DXd are summarized in **Table 1**. Patients, most of whom were female (97.7%, n = 250), had a median (range, minimum–maximum) age of 55 (30–91) years and a median (range) body weight at T-DXd treatment initiation of 65 (39–104) kg. Of the 256 patients, 67.6% (n = 173) had hormone receptor–positive tumors, the majority had a HER2 IHC expression score of 3+ (63.7%, n = 163) or 2+ (30.9%, n = 79), and 39.8% (n = 102) had positive ISH status. All patients had received at least two prior anti-HER2–based regimens and were eligible for the study (see below). Overall, 99.2% of patients (n = 254) received monoclonal antibody HER2 inhibitors, and 53.1% (n = 136) received HER2 tyrosine kinase inhibitors. The intent of anti-HER2 therapy was metastatic therapy in 98.0% of cases (n = 251), adjuvant therapy in 28.5% (n = 73), and neoadjuvant therapy in 18.0% (n = 46). The median number (range) of previous anti-HER2 lines of therapy was 3.0 (0–12) in any setting and 3.0 (0–6) in the metastatic setting (**Table 1**).

**Table 1:** Patient demographics and clinical characteristics.

	All patients (N = 256)*
Median (range) age, years	**55 (30–91)**
Female, n (%)	**250 (97.7)**
Median (range) weight at T-DXd treatment initiation, kg	**65 (39.0–104.0)**
HER2 status, n (%)^†^	
IHC 1+^‡^	3 (1.2)
IHC 2+	79 (30.9)
IHC 3+	163 (63.7)
IHC missing	11 (4.3)
ISH+	102 (39.8)
ISH−	7 (2.7)
ISH N/A	137 (53.5)
ISH missing	10 (3.9)
Hormone receptor status, n (%)	
Positive	173 (67.6)
Negative	78 (30.5)
Missing	5 (2.0)
Site of metastatic disease, n (%)	
Lymph node	143 (55.9)
Bone	140 (54.7)
Liver	107 (41.8)
Lung	104 (40.6)
Brain	85 (33.2)
Other	62 (24.2)
Prior anti-HER2 therapy, n (%)^§^	**254 (99.2)**
Trastuzumab ​	251 (98.0)
T-DM1	219 (85.5)
Pertuzumab​	203 (79.3)
Lapatinib​	117 (45.7)
Neratinib​	20 (7.8)
Tucatinib​	19 (7.4)
Margetuximab​	7 (2.7)
Intent of prior anti-HER2 therapy, n (%)^§^	
Neoadjuvant	46 (18.0)
Adjuvant	73 (28.5)
Metastatic	251 (98.0)
Median (range) lines of prior anti-HER2 therapies in any setting, n^‡^	**3 (0–12)**
Median (range) lines of prior anti-HER2 therapies in metastatic setting, n^‡^	**3 (0–6)**
ECOG PS, n (%)	
0	87 (34.0)
1	83 (32.4)
2	17 (6.6)
3	3 (1.2)
Not done/not documented/unknown	66 (25.8)
Any antiemetic prophylaxis treatment prior to T-DXd initiation, n (%)	**207 (80.9)**
Single-agent regimen	26 (12.6)
Dual-agent regimen	118 (57.0)
Triple-agent regimen	59 (28.5)
Quadruple-agent regimen	4 (1.9)

*Italy (n = 172), Spain (n = 54), and Ireland (n = 30).

^†^HER2 status was determined by a validated method.

^‡^Any patients with entries recorded in the eCRF as being ineligible for the study because of the number of prior anti-HER2 therapies or IHC/ISH status (per the eCRF) were subsequently found to have been eligible for T-DXd treatment through the NPP following a review of medical files after database lock.

^§^If a patient had more than one agent in the same regimen, the patient was counted at each relevant agent. Medications were coded using WHODrug.

ECOG PS, Eastern Cooperative Oncology Group performance status; eCRF, electronic case report form; HER2, human epidermal growth factor receptor 2; IHC, immunohistochemistry; ISH, *in situ* hybridization; N/A, not available; NPP, named patient program; T-DM1, trastuzumab emtansine; T-DXd, trastuzumab deruxtecan; WHO, World Health Organization.

Bold text rows indicate a top-level category and non-bold text rows beneath represent associated subcategories.

Based solely on the information submitted in the eCRFs, nine patients did not meet the inclusion criteria (three patients for not having received ≥2 prior anti-HER2–based regimens and six patients for not meeting the inclusion criterion of ‘documented HER2-positive tumor status by a validated method’). However, upon reviewing their medical files after the database lock, all nine patients were found to have met the eligibility criteria outlined in the patient access forms of the NPP. These nine patients were included in the full analysis set but excluded from a *post-hoc* sensitivity analysis of rwTTD and rwPFS, which was conducted to evaluate the potential impact of these apparent protocol deviations.

### 3.2 Patient disposition

Patient disposition is summarized in **Table 2**. At the time of data cutoff (March 28, 2024), 5.1% of patients (n = 13) were still receiving T-DXd treatment under the NPP. In total, 94.9% of patients (n = 243) had discontinued T-DXd treatment under the NPP. The main reasons for discontinuation included disease progression (46.1%, n = 112) and patients switching to commercial supplies of T-DXd (30.0%, n = 73). The median (range) follow-up duration was 11.9 (0.0–33.7) months. Overall, 39 deaths were reported; of these, 94.9% (n = 37) were related to disease.

**Table 2:** Patient status, follow up, and outcomes.

	N = 256
T-DXd ongoing under the NPP at EUROPA T-DXd study end, n (%)	**13 (5.1)**
T-DXd discontinued under the NPP, n (%)	**243 (94.9)**
Disease progression	112 (46.1)
Patient switched to commercial supplies	73 (30.0)
Other	25 (10.3)
Death	16 (6.6)
Adverse event	15 (6.2)
Patient decision to stop T-DXd treatment	2 (0.8)
Median (range) duration of follow up, months	**11.9 (0.0–33.7)**
Median (range) total treatment duration, months*	**11.2 (0.0–30.4)**
Median (range) number of cycles of T-DXd received	**15.0 (1–41)**
Total deaths, n (%)^†^	**39 (15.2)**
Disease related	37 (94.9)
Non-disease related	2 (5.1)

*Treatment duration was calculated as (date of the last dose – date of treatment initiation)*12/365.25. For patients still ongoing, and with missing last dose date, data cutoff date was used as the last dose date.

^†^Includes patients who discontinued T-DXd under NPP prior to death.

NPP, named patient program; T-DXd, trastuzumab deruxtecan.

Bold text rows indicate a top-level category and non-bold text rows beneath represent associated subcategories.

### 3.3 Outcomes

**Figure 2:**
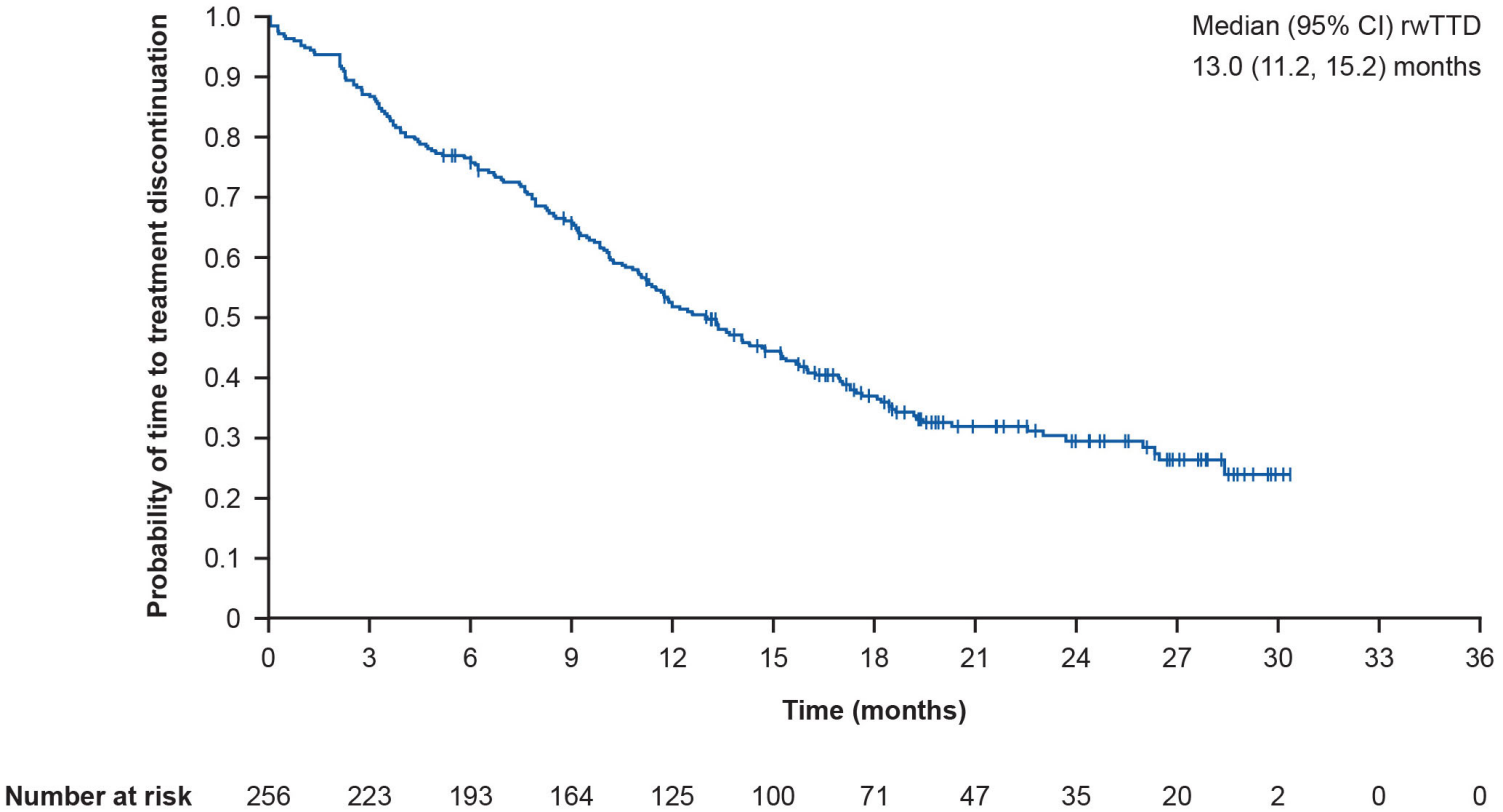
Kaplan-Meier curve showing overall rwTTD. rwTTD is defined as time from T-DXd initiation date to time of T-DXd discontinuation for any reason; CI for median was computed using the Brookmeyer and Crowley method; vertical marks indicate censored data to the time of T-DXd discontinuation for any reason. CI, confidence interval; rwTTD, real-world time to treatment discontinuation; T-DXd, trastuzumab deruxtecan.

Among all 256 patients who received T-DXd, the primary endpoint of median (95% CI) rwTTD was 13.0 (11.2, 15.2) months (**Figure 2**). Following *post-hoc* sensitivity analyses, the median (95% CI) rwTTD was 13.3 (11.3, 15.3) months. In prespecified subgroup analyses, the median (95% CI) rwTTD was 12.5 (10.2, 17.3) months in patients with brain metastases and 13.3 (10.6, 15.8) months in patients without brain metastases. In patients with liver metastases, the median (95% CI) rwTTD was 10.0 (8.5, 11.4) months versus 16.0 (13.0, 18.4) months in patients without liver metastases. Furthermore, median (95% CI) rwTTD was 13.7 (10.0, 18.4) months versus 12.2 (11.0, 15.3) months in patients with and without lung metastases. In patients with and without lymph node metastases, median (95% CI) rwTTD was 14.8 (11.3, 17.5) months versus 11.7 (9.5, 14.1) months. The median (95% CI) rwTTD in patients with and without bone metastases was 11.0 (9.1, 13.4) months versus 15.2 (12.2, 18.2) months.

**Figure 3:**
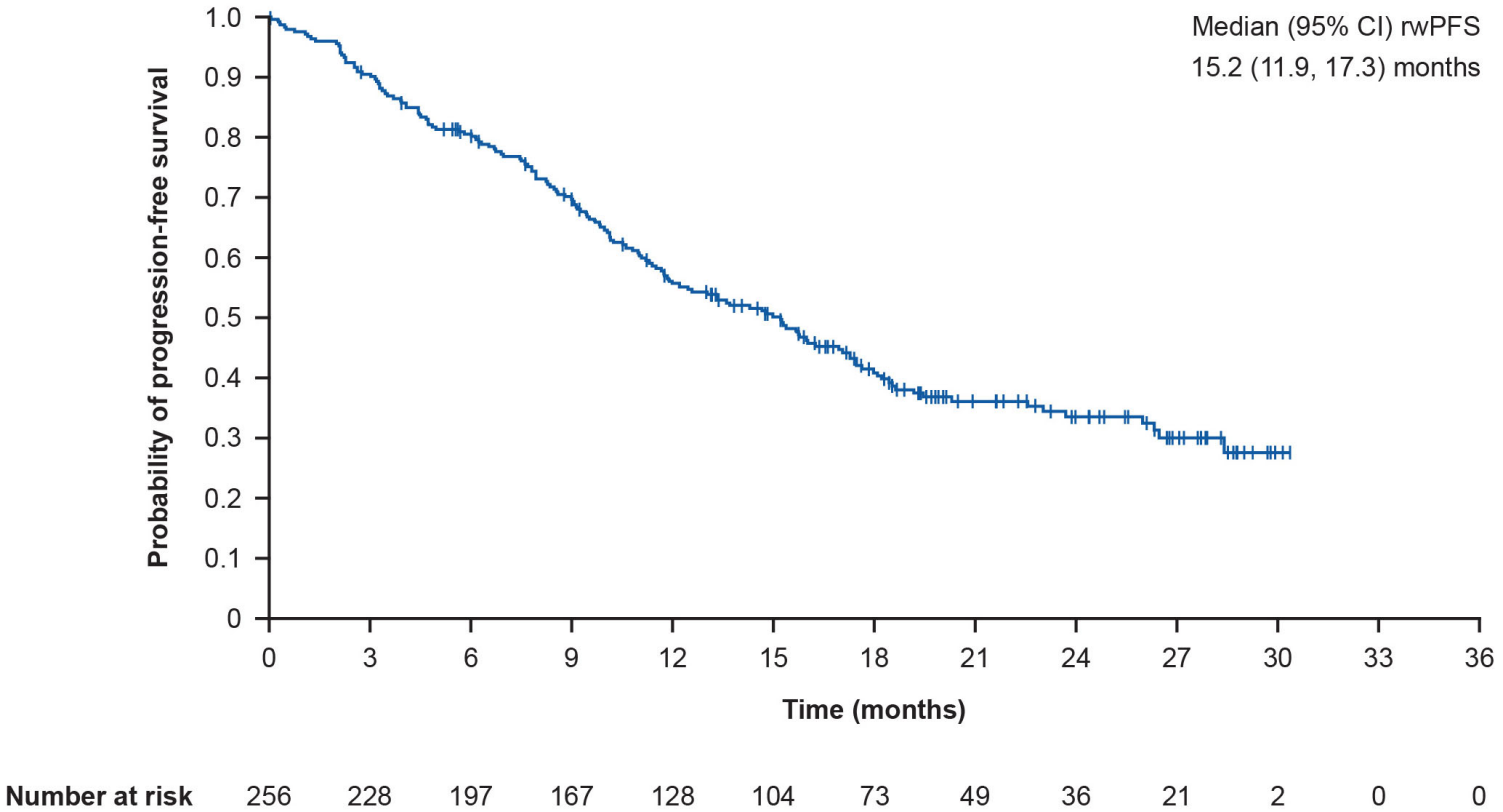
Kaplan-Meier curve showing overall rwPFS. rwPFS is defined as time from T-DXd initiation to the earliest of death or progression; CI for median was computed using the Brookmeyer and Crowley method; vertical marks indicate censored data to the time of an event. CI, confidence interval; rwPFS, real-world progression-free survival; T-DXd, trastuzumab deruxtecan.

The overall median (95% CI) rwPFS was 15.2 (11.9, 17.3) months (**Figure 3**). Following *post-hoc* sensitivity analyses, the median (95% CI) rwPFS was 15.2 (11.9, 17.3) months. In prespecified subgroup analyses, the median (95% CI) rwPFS was 14.8 (11.1, 26.5) months in patients with brain metastases, and 15.2 (11.4, 17.5) months in patients without brain metastases. Furthermore, the median (95% CI) rwPFS was 11.0 (9.4, 13.6) months in patients with liver metastases versus 17.3 (15.0, 19.2) months in patients without liver metastases. In patients with versus without lung metastases, median (95% CI) rwPFS was 15.7 (10.6, 26.0) months versus 14.8 (11.4, 17.3) months. In patients with and without lymph node metastases, median (95% CI) rwPFS was 17.0 (12.0, 19.2) months versus 13.6 (10.1, 15.8) months. Finally, median (95% CI) rwPFS was 11.3 (9.4, 15.7) months versus 17.1 (14.3, 20.3) months in patients with and without bone metastases, respectively.

In exploratory analyses, median overall survival was not mature at data cutoff and could not be estimated. Kaplan-Meier analysis demonstrated 6-, 12- and 18-month overall survival rates (95% CI) of 91.3% (87.0%, 94.3%), 84.9% (79.3%, 89.1%), and 82.2% (75.7%, 87.0%), respectively.

### 3.4 Safety

Prior to T-DXd initiation, use of an antiemetic regimen with prophylactic intent was reported in 80.9% of patients (n = 207) in total, of whom most were treated with dual (57.0%; n = 118) or triple (28.5%; n = 59) regimens. Of the 256 patients, TEAEs were reported via the pharmacovigilance system in 33.2% of patients (n = 85), and serious TEAEs were reported in 9.4% (n = 24) (**Table 3**). Nausea (7.0%; n = 18) was the only TEAE reported in over 5% of patients. TEAEs led to the discontinuation of T-DXd in 12.9% of patients (n = 33) and a dose interruption in 4.3% (n = 11). Overall, 2.7% of patients (n = 7) had interstitial lung disease (ILD), as reported by physicians (Grade ≤2, n = 5; T-DXd discontinuation, n = 3; dose interruption, n = 1); pneumonitis was reported in 3.5% of patients (n = 9; T-DXd discontinuation, n = 3; dose interruption, n = 5). Additionally, neutropenia and a decreased ejection fraction were reported in 2.3% (n = 6) and 0.8% (n = 2) of patients, respectively.

**Table 3:** Summary of safety and tolerability in the overall population.

n (%)	N = 256
Any TEAE	85 (33.2)
Serious TEAEs	24 (9.4)
TEAEs leading to T-DXd interruption	11 (4.3)
TEAEs leading to T-DXd discontinuation	33 (12.9)
TEAEs leading to death	15 (5.9)

*To comply with pharmacovigilance legislation, physicians treating under the NPP reported serious and non-serious AEs and/or safety information using an AE report form and targeted questionnaires.

^†^The collection of TEAE data via the NPP included standard terms from the MedDRA; any preferred terms captured in the NPP that related to disease progression and metastases were omitted from this report: metastases to central nervous system, metastases to liver, metastases to bone, metastases to lymph nodes, metastases to lung, metastases to breast, metastases to peritoneum, metastases to pleura, metastases to skin, breast cancer, breast cancer metastatic, metastases to chest wall, metastases to meninges, metastases to soft tissue, metastases to the mediastinum, metastatic neoplasm, and disease progression.

AE, adverse event; MedDRA, Medical Dictionary for Regulatory Activities; NPP, named patient program; T-DXd, trastuzumab deruxtecan; TEAE, treatment-emergent adverse event.

During data collection, 5.9% of patients (n = 15) were recorded as having TEAEs that led to death (**Table 3**). The majority were recorded as being related to disease or worsening of general condition. Other TEAEs leading to death were recorded as ILD/pneumonitis (n = 2), necrotizing fasciitis with concurrent neutropenia and septic shock (n = 1), myocardial infarction (n = 1), and cardiac death (n = 1).

## 4 Discussion

This is the first large RWD analysis of T-DXd use in patients from different European countries and confirms that the data generated in phase II/III clinical trials in the third-line setting and beyond for HER2-positive metastatic breast cancer apply in real-world clinical practice. In particular, rwTTD and rwPFS were broadly consistent with data from T-DXd arms of clinical trials (DESTINY-Breast01 and DESTINY-Breast02) (16, 19), confirming the effectiveness of T-DXd in heavily pretreated patients: median (95% CI) rwTTD was 13.0 (11.2, 15.2) months and median (95% CI) rwPFS was 15.2 (11.9, 17.3) months. In the subset of patients with brain metastases, the median (95% CI) rwPFS was 14.8 (11.1, 26.5) months, and in patients without brain metastases, the median (95% CI) rwPFS was 15.2 (11.4, 17.5) months.

Differences in study eligibility criteria should be noted. In EUROPA T-DXd, baseline brain imaging was not mandatory, and patients with stable/treated and progressive/untreated brain metastases were eligible. In DESTINY-Breast02, baseline brain imaging was required for all patients during the screening period, and only patients with clinically inactive brain metastases and treated asymptomatic brain metastases not requiring corticosteroids or anticonvulsants were eligible (19). More recently, results from the phase IIIb/IV DESTINY-Breast12 study evaluating the efficacy and safety of T-DXd in patients with HER2-positive metastatic breast cancer with and without baseline brain metastases were reported (30). In patients with baseline brain metastases (previously treated stable brain metastases and active [untreated or previously treated and progressing] brain metastases), the median (95% CI) progression-free survival was 17.3 (13.7, 22.1) months (30).

Despite positive efficacy outcomes, the overall rate of discontinuation in EUROPA T-DXd was notably high (94.9%). It is important to recognize that the discontinuation rate included 30% of patients who did not continue in the NPP when T-DXd became commercially available in their country. This is a unique consequence of the study design that does not reflect the expected rate of discontinuation from T-DXd in real-world clinical practice. The required censoring of these patients may have decreased the precision of the estimates obtained and contributed to rwTTD being shorter than rwPFS. Nevertheless, the median rwTTD and rwPFS are consistent with the estimates reported in clinical trials and add to the evidence supporting use of T-DXd in previously treated patients with HER2-positive metastatic breast cancer with or without brain metastases at baseline, while strengthening the notion that this censoring does not have an impact on the results.

Although the current results provide reassurance that RWD with T-DXd reflect those of clinical trials, EUROPA T-DXd was an observational study based on the collection of a limited set of data points from an NPP; therefore, comparisons with carefully controlled interventional trials are indicative only because of differences in patient populations and the conditions under which treatment is given and monitored. However, it is relevant to note that the baseline characteristics and demographics of patients in EUROPA T-DXd were broadly aligned to those in the T-DXd arm of the DESTINY-Breast clinical trials (16, 19); for example, in EUROPA T-DXd, the median patient age was 55.0 years, 67.6% of patients had hormone receptor–positive tumors, and 63.7% of patients had a HER2 expression score of 3+. The median number of previous lines of therapy for metastatic disease was three in EUROPA T-DXd, six in DESTINY-Breast01 (16), and two in DESTINY-Breast02 (19). This suggests that patients who were recruited to these clinical trials are broadly representative of those who were being prescribed T-DXd in routine clinical practice, which supports the expectation of similar antitumor effects.

Our data are also consistent with other published, retrospectively collected, RWE on T-DXd. In the DE-REAL study of 143 patients with HER2-positive metastatic breast cancer treated at 12 Italian hospitals, rwPFS was 16 months (31). In an interim analysis of REALITY-01, an RWE study of patients who received T-DXd through an early access program or after marketing authorization following at least two lines of prior therapy for metastatic/unresectable HER2-positive breast cancer, median rwPFS was 17.4 months (32). A further retrospective study using data from electronic health records on the Flatiron Health platform reported a median rwPFS of 12.1 months in patients with HER2-positive metastatic breast cancer (33).

In EUROPA T-DXd, the most reported TEAE was nausea (7%); 9.4% of patients had serious TEAEs, and 2.7% experienced ILD. In DE-REAL, the most frequent AEs were nausea (in 33% of patients, in contrast to the current study in which prophylaxis was employed in 80.9% of patients), neutropenia (21%), and asthenia/fatigue (21%); 18% of patients had severe AEs, and 2% experienced ILD, while AE-related dose reduction occurred in 26% of patients but did not affect response to treatment or outcomes (31). In REALITY-01, serious AEs occurred in 9.5% of patients, and AE-related dose reduction was reported in 14.4% of patients; 14.1% of patients had an ILD event (32).

Patients were treated for a median (range) of 15 (1–41) cycles and had a median total treatment duration of 11.2 months. Excluding patients who switched to a commercial T-DXd supply, the discontinuation rate was 66.4%. For comparison, a real-world UK patient population with HER2-positive metastatic breast cancer reported T-DXd discontinuation in 55% of patients (median [range] treatment duration 9 [1–28] cycles) (34). Discontinuation related to AEs was reported in 3% and 10.2% of patients in the DE-REAL (median follow-up duration 12 months) and REALITY-01 (median follow-up duration 17.7 months) studies, respectively (31, 32), compared with 6.2% in the current study (median follow-up duration 11.9 months).

This study provides a robust evaluation of real-world outcomes for a selected group of patients who received T-DXd under the NPP for HER2-positive metastatic breast cancer. However, it is associated with limitations:

*Selection bias:* there is likely to have been a selection bias in favor of large, high-enrolling NPP centers associated with an academic/research setting, and patients who experienced rapid disease progression following multiple prior lines of treatment, including T-DXd, may have been less willing to participate. Consequently, the validity and degree of generalizability of the findings may be affected.

*Data completeness:* data relied on accurate retrospective reporting via eCRF entry owing to the inclusion of patients who had already completed therapy or were receiving ongoing therapy; the inclusion of patients with missing or incomplete eligibility data highlights the potential for compromised data reliability. For example, the full analysis set included patients with inaccurate eCRF records for prior anti-HER2 therapies, or with missing IHC/ISH status. Furthermore, receipt of prior anti-HER2 therapies was only mandated in the metastatic setting after the study had been initiated, meaning that some patients may have met the eligibility criteria based on the receipt of two or more prior anti-HER2 therapy regimens in (neo)adjuvant settings. To mitigate the effects of missing data in the eCRFs, patient medical files were reviewed following database lock. Accordingly, it was confirmed that, as intended, the vast majority of patients in this study had IHC 3+ or 2+ status and received two or more prior anti-HER2 therapies in the metastatic setting. Sensitivity analyses were performed, excluding patients with ineligible prior anti-HER2 therapy or IHC/ISH status (per the eCRF); no differences were observed.

*Safety reporting:* the robustness and reliability of the safety findings may be affected by under or inconsistent reporting of safety events owing to the use of a pharmacovigilance system, as opposed to standardized clinical trial methodologies. Therefore, conclusions about the safety profile should be interpreted with caution.

*Patient follow up:* data collection was restricted to T-DXd treatment provided under the terms of the NPP, so data could not be collected from any patients who switched to commercially available T-DXd treatments owing to the drug becoming approved in the relevant territory. This resulted in high censoring rates for the efficacy endpoints. Increases in real-world use of T-DXd, plus accumulating exposure in earlier lines of therapy, will enable additional RWD analyses to expand upon the findings of this study.

In conclusion, results from the real-world EUROPA T-DXd study demonstrate that the effectiveness and tolerability of T-DXd in heavily pretreated patients with HER2-positive metastatic breast cancer in the clinical practice setting are consistent with data reported in clinical trials. These findings provide further support for T-DXd as a treatment option in the real-world clinical setting. There is an ongoing need for RWD collection studies as T-DXd is approved for treatment more widely, including in earlier lines of therapy.

## Data Availability

The datasets presented in this study can be found in online repositories. The names of the repository/repositories and accession number(s) can be found below: Data underlying the findings described in this article may be obtained in accordance with Daiichi Sankyo’s data-sharing policy, which is described at https://www.daiichisankyo.com/rd/clinical_trials/. Data for studies directly listed on Vivli can be requested through Vivli at www.vivli.org. Data for studies not listed on Vivli can be requested through Vivli at https://vivli.org/members/enquiries-about-studies-not-listed-on-the-vivli-platform/. The Daiichi Sankyo Vivli member page is also available, outlining further details: https://vivli.org/ourmember/daiichi-sankyo/.
